# Underutilized treatments for patients with refractory cancer pain: a qualitative study assessing the use of intrathecal drug delivery devices in the United Kingdom compared to alternative treatments in cancer pain management

**DOI:** 10.3389/fpain.2025.1481245

**Published:** 2025-02-20

**Authors:** Victoria Barnosky, Matthew Brown, Somnath Bagchi, Remy Blain, Demir Husejnovic, Sandra Johnson, Meredith Mackworth-Praed

**Affiliations:** ^1^ School of Health Professions, Robert Morris University, Pittsburgh, PA, United States; ^2^ Health Economics Outcomes Research, Suazio Consulting, Antwerp, Belgium; ^3^ Pain Medicine Department, The Royal Marsden Hospital, and Honorary Associate Faculty, The Institute of Cancer Research, London, United Kingdom; ^4^ Chronic Pain Unit, Derriford Hospital, Plymouth, United Kingdom

**Keywords:** cancer, refractory pain, pain interventions, qualitative study, intrathecal drug delivery

## Abstract

**Objectives:**

This research aims to better delineate how intrathecal drug delivery systems (IDDS) are incorporated into the oncology care continuum and highlight the need for further awareness of interventional options for pain management of cancer patients in the United Kingdom. The study focuses on exploring the knowledge, perspectives, and experiences of healthcare professionals regarding IDDS as a treatment option for managing chronic refractory pain in cancer patients.

**Methodology:**

A thematic coding using inductive analysis was employed to achieve the research objectives. Semi-structured interviews were conducted with 18 healthcare professionals in various specialties, including oncology, neurosurgery, pain management, and palliative care. The interviews were transcribed, and a two-phased qualitative inductive coding approach was used to analyze the data.

**Results:**

The findings of the study revealed four major themes: Education, Barriers & Benefits, Technical & Administrative, and Patient-Centered Care. The theme of Education highlighted the need for increased knowledge and awareness of IDDS among healthcare professionals. Barriers & Benefits encompassed concerns about infection risk, suitability for patients with a short life expectancy, and the challenges and advantages of IDDS use. The Technical & Administrative theme addressed cost considerations, device management, and the need for improved guidelines. Patient-Centered Care emphasized the importance of involving patients in decision-making and considering their physical and emotional well-being throughout the treatment pathway.

**Conclusions:**

This research identifies several areas of unmet need in the management of refractory pain in cancer patients, including the development of more inclusive guidelines, greater awareness among clinicians and patients, and the role of medical technology companies in supporting effective pain management. The findings underscore the impact of IDDS on improving pain control and highlight the potential importance of early intervention and comprehensive pain management in influencing the trajectory of oncological diseases.

## Introduction

Pain, related to cancer, that is refractory to standard pharmacological treatment is a common and debilitating symptom in patients with cancer that can significantly impact their quality of life (1). Oncology patients face many physical, emotional, spiritual and psychological challenges during their cancer journey, and poorly controlled pain has significant negative consequences for them. Refractory pain is defined as pain not responding to standard pharmacological treatments, this situation occurs in approximately 10%–20% of oncology patients.

This research aims to understand how intrathecal drug delivery pumps are incorporated into the oncology care pathway and to highlight the need for increased awareness of advanced interventional options for pain management in the United Kingdom.

The use of intrathecal pumps to deliver analgesic drugs in cancer patients with chronic refractory pain has gained increasing attention over the past decade. Although intrathecal pain pumps are an effective option for the management of pain in these patients, many clinicians in the UK are still unaware of the benefits or unsure about the process to facilitate the implantation of an intrathecal pump in their patients (2).

## Literature review

### Refractory pain

Adequate pain control is one of the most common challenges and one of the biggest concerns of patients with cancer. The World Health Organization has a three-step analgesic ladder that is recognized as the gold standard for pain management and is effective in 70%–90% of patients (3, 4). The remaining 10%–30% of patients who do not respond to the measures recommended will experience pain that is refractory to common medication-based treatments. A recent UK national patient cancer survey indicated that 18% of patients with cancer perceive that their pain is not managed (5).

Refractory pain is characterized by a resistance to conventional pain management strategies, including the use of oral opioids, adjuvant medications, and non-pharmacological interventions. Several factors may result in refractory pain in patients, these include tissue destruction by tumor, nerve involvement, inflammation, and treatment-related complications. Patients often experience severe pain that hinders their ability to perform daily activities, disrupts sleep patterns, and causes emotional distress. A recent pan-European survey of patients with all stages of cancer identified that 69% had functional levels impacted by pain (6). Managing refractory pain in cancer patients presents a considerable clinical challenge and consumes significant healthcare resources. Therefore, there is a substantial need for advanced therapeutic approaches, including intrathecal drug delivery devices, to provide a further option for effective analgesia. Studies in both Europe and the United States show that pain at any stage of cancer is commonly treated inadequately with between 56 and 82.3% of patients shown to not receive sufficient pain relief (7). Additionally, the overall cancer survival rates have been increasing across the world. Even during the Covid-19 pandemic while healthcare organizations were challenged to maintain patient care, the 2020 overall cancer survival rate for those in the United Kingdom increased by 9% growing to 74.6% (8). As more patients survive a diagnosis of cancer for long periods of time, the number of patients experiencing long-term pain related to either their disease or its treatment also increases, requiring more and more ambulatory patients to request pain management to continue with activities of daily living (9).

### Guidelines for pain management

Many specialty-specific societies, regulators and governmental bodies across the globe produce guidelines, recommendations and standards for cancer pain management in an attempt to ensure optimal patient care. These resources are intended to support clinicians and disseminate uniform best practices among healthcare professionals. Examples include those from the National Institute for Health and Care Excellence (NICE), the European Society of Anaesthesiology (ESA), the UK's Faculty of Pain Medicine and the European Association for Palliative Care (EAPC). Although these are guidelines and therefore compliance is not mandated, societies encourage clinicians to adopt these recommendations into their practice to ensure that the management of pain is consistent and in line with current evidence. A common feature of these guidelines is a recommendation for an escalation-based approach to treating patients with cancer who are experiencing pain. The first step of the approach commonly includes non-opioid medications, followed by opioids, then strong opioids which are administered until the pain is controlled (10). In general, the guidelines are broad enough that deviation from the suggested best practice may take place. Considerations such as patient preference, contraindications or resource availability may be factors when considering pain management approaches. For example, the current WHO guidelines do not include interventional approaches to pain management or include the challenges of using systemic opioids (10, 11).

### Oral, topical, and injection/infusion routes of administration

Analgesic medication can be delivered by a number of routes. Decisions regarding the route selected may be influenced by individual patient characteristics, for example, a patient's ability to swallow will directly influence the pharmacokinetic properties of the drug.

Strong opioids remain the standard treatment for moderate to severe cancer pain and morphine is the most commonly used opioid in these situations. Morphine is usually administered orally but can be given intravenously or subcutaneously, generally if more rapid analgesia is required. Oxycodone, hydromorphone, and methadone can be used as alternative strong opioids. Some patients may also find relief from transdermal fentanyl or buprenorphine patches. Transdermal administration ensures a consistent dose of analgesic and reduces the requirement for frequent IV or oral administration.

A common feature of the continued use of opioids is the development of opioid tolerance, which decreases the effect of the medication. If a patient develops this phenomenon, it is recommended to switch or rotate treatment to alternative opioids to continue pain management (12). Patients who are unable to receive opioids orally or via the transdermal route can receive them subcutaneously and if this route of administration is not possible, or immediate pain relief is needed for severe pain, intravenous administration can be considered.

Even with the intent to follow a systematic guideline for pain management, clinicians and patients may face challenges when administering oral or intravenous pain medication. Cognitive impairment, drowsiness, and constipation can deter the use of pain medication. Various studies have shown instances of constipation ranging from 40% to 68% in patients being treated with opioids (13, 14). 30%–60% of patients experience moderate to severe fatigue throughout a cancer diagnosis due to multiple factors including the effect of the cancer, chemotherapy or radiotherapy, however, fatigue resulting from opioids or other analgesics may also contribute. The data available today is currently limited but is seeking to better understand the impact of how these drugs negatively impact cancer-related fatigue (15). Recent evidence suggests the Mu receptor has a profound role in cancer progression and systemic opioids promote it (16).

### Intrathecal drug delivery—the mechanism and current commissioning in the UK

**Figure 1:**
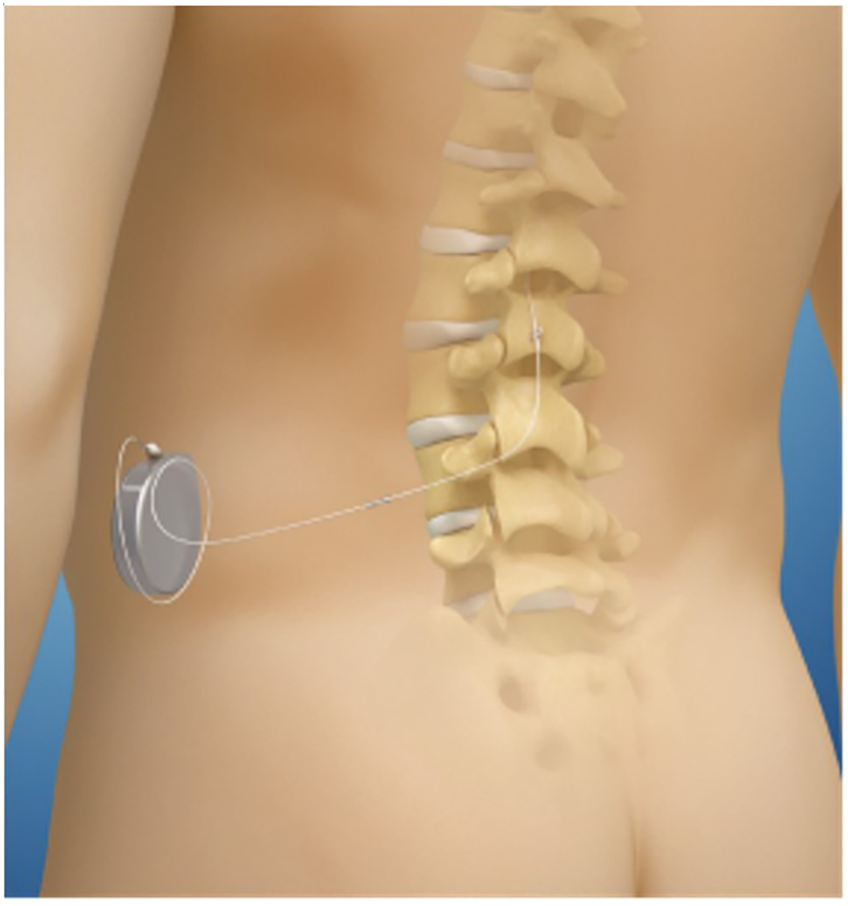
Schematic of anatomical location of implanted pump and catheter in reference to spinal column and cord.

Intrathecal drug delivery systems (IDDS) also known as intrathecal pumps, are surgically implanted devices. A continuous infusion of analgesics is delivered via a tunneled catheter, through the dura into the cerebral spinal fluid (CSF). Figure 1 the dorsal horn of the spinal cord within the intrathecal space is a primary site for pain processing, therefore various receptors in this area can be targeted to provide analgesia. By targeting opioid, GABA, alpha-2, or dopaminergic receptors with medication introduced directly into the CSF, the need for the drug to cross the blood-brain barrier is eliminated. This results in the requirement for lower amounts of medication to achieve an analgesic response (17, 18) and a reduction in the potential for systemic side effects to occur. Additionally, the intrathecal route permits a more rapid targeting of the pain processing sites when compared to alternative routes of administration, resulting in an accelerated clinical response (18).

The National Health Service (NHS) in the UK recognizes pain that is refractory to standard-of-care analgesic drugs can cause significant physical and emotional distress for patients and their caregivers. The NHS supports the use of IDDS devices when other forms of pain management have failed and the British Pain Society asserts that intrathecal drug delivery can be “an effective method of pain control with supporting evidence for chronic non-malignant pain, pain associated with cancer, and spasticity (19, 20)”. A recent study out of Leeds shows moderate evidence obtained from two separate randomly controlled trials to consider epidural or intrathecal opioids for patients with end-stage cancer pain (21).

## Methodology

### Approach

The design of this qualitative study leveraged semi-structured interviews to understand how members of the oncology and pain management teams recognize and use intrathecal drug delivery systems as part of their treatment approach for patients experiencing refractory cancer pain. The focus of the interviews was two-fold, concentrating firstly on the current use of IDDS in clinical practice and secondly how healthcare professionals perceive the use of this technology in a broader environment. The ultimate intent was to employ inductive coding to analyze the content collected during each interview.

Qualitative research using inductive content analysis is a common methodology used in healthcare when the sample size of the study is relatively low, but many details need to be extrapolated from the findings (22). Qualitative coding, also referred to as “thematic analysis,” is a process used to systematically categorize spoken dialogue into themes or patterns. The thematic analysis allows the researcher to understand and quantify a deeper meaning and specific concepts of the discussion, not just simply answers to the questions being asked (23). In contrast to deductive coding where themes are predefined before the research begins, inductive coding is a ground-up approach where the coding themes are determined by the data itself. This type of approach reduces any preconceived notions and allows the findings to emerge from the raw data, creating less bias. Phase 1 of the data coding identifies categories for each statement being made during the interview. Once those specific categories are created, phase 2 coding places those items into broad themes. The broad themes, hence, create the overall structure for interpreting and presenting the findings. This allows the researcher to present a broad theme but then drill down deeper into the underlying phase 1 codes included in that theme.

### Recruitment

Study participants were recruited from a range of clinical roles including the specialties of Oncology, Neurosurgery, Pain Management, and Palliative Care to participate in a 60-minute double-blinded interview conducted by a third-party research company. Participants were recruited from across the United Kingdom and were paid a financial honorarium of fair market value for their participation in the study. Potential participants were screened based on the following criteria: their clinical role, the number of years they have been working in their current position, and their familiarity with IDDSs. Inclusion criteria were based on obtaining a mix of roles, experience, and both current users of IDDSs and non-IDDS users. Additionally, demographic information was collected to include the participants’ annual patient volume, types of treatments for refractory pain, and current knowledge of pricing for various pain management techniques.

### Participants

In total, the interviews from eighteen participants were included in the analysis (Table 1). All participants provided informed consent (see Declarations for more information on informed consent). The participants can be categorized into the roles of Oncology Consultants (8), Palliative Pain Consultants (2), Neurosurgeons (2), Nurses (4), and Service Managers (2). Their experience in their current role ranged from two to thirty years with an average of 12.2 years. Twelve out of the eighteen participants were employed by a university-based organization while six considered their facility to be a district or general hospital. Based on the recruiting objective, the number of patients currently being treated for refractory pain covered a broad range from four patients to over one hundred patients currently in their care.

**Table 1:** Description of study participants recruited from across the United Kingdom including the specialties of oncology, neurosurgery, pain management, and palliative care, who participated in the double-blinded interview.

Participant	Role	Experience (in years)	# of patients currently in treatment for refractory pain
Participant #1	Palliative Care Consultant	5	30
Participant #2	Oncologist	25	60
Participant #3	Pain Specialist/Neurosurgeon	2	25
Participant #4	Service Manager	5	10
Participant #5	Nurse	10	30
Participant #6	Pain Specialist/Neurosurgeon	25	15
Participant #7	Nurse	30	10
Participant #8	Nurse	2	5
Participant #9	Nurse	10	4
Participant #10	Service Manager	14	30
Participant #11	Oncologist	7	25
Participant #12	Oncologist	5	20
Participant #13	Palliative Care Consultant	10	4
Participant #14	Oncologist	20	10
Participant #15	Oncologist	13	120
Participant #16	Oncologist	21	100
Participant #17	Oncologist	9	70
Participant #18	Oncologist	7	100

### Study execution

The study did not meet the NHS Health Research Authority (HRA) requirement for ethical approval as all participants willingly volunteered and anonymity was maintained; however, data privacy was preserved in compliance with GDPR and stored in accordance with ISO 27001 throughout the duration of the research and during post-study data storage. Once individuals were determined to meet the screening criteria for participation, they were scheduled to participate in a semi-structured interview. The interviews were conducted over the Microsoft Teams platform between January and December of 2022 by a senior member of the outcomes research team with training in inductive coding and analysis. At the start of each interview, participants were presented with the topics being discussed and explained the benefits and risks of participating in the study. Participants were required to give verbal authorization to record the interview for transcription purposes.

### Study analysis

Immediately after each interview, the recordings were transcribed by a member of the research team, and quality checks were performed to ensure accuracy. Once all interviews were completed and transcribed, a two-phased qualitative coding approach was taken using inductive analysis. The process of deriving themes followed a rigorous two-phase inductive coding approach to ensure the findings were grounded in the raw data. In Phase 1, line-by-line coding was performed on the interview transcripts to generate initial codes. These codes represented discrete ideas or observations directly articulated by participants. The coding team comprised two researchers with expertise in qualitative methods, who worked independently to minimize bias. Discrepancies between initial codes were resolved through iterative discussion, resulting in a comprehensive set of mutually agreed-upon categories. In Phase 2, the codes were systematically grouped into broader themes based on conceptual similarities. This thematic grouping was guided by a constant comparative method, where emerging themes were compared against the full dataset to ensure they captured the breadth and depth of participant perspectives.

To validate the robustness of the themes, member checking was employed, where subject matter experts reviewed the preliminary thematic structure to confirm its accuracy and relevance. Feedback from the clinical experts reinforced the clarity and representativeness of the themes. Additionally, the research team conducted peer debriefing sessions to review and refine the thematic structure, ensuring that it remained unbiased and reflective of the data. Finally, thematic saturation was achieved, as no new codes or themes emerged from the data after analyzing the transcripts. This comprehensive and transparent approach to deriving and validating themes enhances the credibility and trustworthiness of the findings, ensuring they reflect the lived experiences and insights of the healthcare professionals interviewed.

The two-phased inductive analysis was completed using Microsoft Excel.

## Findings

Phase 1 coding produced 356 unique categories of topics across all interviews. After phase 2 coding and analysis, those 356 unique categories were placed into four themes or concepts with sixteen subcategories of data (Table 2). The themes that emerged from the interviews were: Education, referring to the knowledge and best practices for managing refractory pain intrathecally; Barriers & Benefits, understanding the indications and contraindications for intrathecal pain devices; Technical & Administrative, the requirements for device management, cost implementations, and regulations; and Patient-Centered Care, understanding the impact on the patient at each stage of the care pathway.

**Table 2:** Summary of the phase 2 coding that resulted in four themes and sixteen subcategories.

Themes/concepts	Subcategories	
Education	Adoption: - Barriers that impact adoption of new pain control methods, including IDDS.	
	Awareness: - Lack of structured guidelines directedly focused on IDDS. - Approach to evaluating and measuring refractory pain.	Availability: - Having greater access to information about IDDS.
Barriers & benefits	Barriers: - Current guidelines do not present a standard life expectancy to qualify for an implantable pain device. - Process to refer patients.	Complications: - Risk of infection in patients who may already be compromised due to cancer treatment.
	Benefits: - differing perceptions of the benefits of those treating patients with IDDS compared to those who do not use this technology.	
	Challenges: - Weighing the benefits vs. the possible risks for the patient receiving IDDS. - Determining the specialty that is responsible for the patient pathway.	
Technical & administrative	Cost: - Impact of the cost of IDDS compared to other treatment options.	
	Guidelines: - Limitations of the current guidelines.	Definition: - Establish standardized criteria for assessing refractory pain.
	Device management: - Logistical aspects of managing devices after implant.	
Patient-centered care	Care pathway: - Pathway for refractory pain management is not as clearly defined as for other diagnoses.	
	Treatment strategy: - Outline the strategy of controlling pain vs. controlling the cancer resulting in pain.	
	Patient: - Patients are the main decision maker throughout the treatment pathway.	Physical: - Concerns about body dysmorphia.
		Emotional: - Concerns about the psychological impact of having a foreign body implanted.

### Education

The first concept that emerged during the analysis of this study focused on education. Many of the study participants did not understand the use of intrathecal pumps for the management of refractory pain in oncology patients. Apart from those who were currently managing patients’ pain using intrathecal pumps (*n* = 9), clinicians identified a need for more education regarding this type of care management.

The approach to evaluating and measuring refractory pain differed among clinicians. Although all participants agreed that refractory pain is defined as pain that cannot be adequately controlled despite aggressive measures, the criteria for evaluating the patient's pain varied. When considering specialist society or best practice guidelines, more than half of the participants quoted the National Institute for Health and Care Excellence (NICE) guidelines. Although these are not intended to be used in specialist care of complex patients.

When assessing pain using a pain score, such as the 11-point NRS (Numeric Pain Rating Scale), participants indicated that this is the patient's perception and should be used along with general assessments of the patient. Although only 38% (7 out of 18) participants were inclined to use a maximum opioid dosage level as an indicator of refractory pain, 100% stated that breakthrough pain was an indicator of a patient's pain level.

Education approaches can be further defined as techniques of adoption and overall awareness of the methods. Environment barriers such as the disruption which occurred in health systems during the Covid-19 epidemic or staffing challenges that resulted afterward have overshadowed and possibly deprioritized the adoption of new pain control methods, including IDDS. These external factors have resulted in avoidance amongst both users and non-users of intrathecal pain management devices. Quoting a palliative care consultant who previously recommended IDDS for his patients: “Due to covid, this procedure has too many delays to be prescribed to my patients”.

Along with challenges with the adoption of IDDS, clinicians identified a lack of structured guidelines that are directedly focused on intrathecal devices. NICE, EAPC, ESA, ASCO, and regional recommendations were identified but were more directed to general pain management approaches or medication selection rather than the route and device used. The participants who were not currently using IDDS in their practice commonly reported a need for more knowledge and best practices.

### Barriers and benefits

The next theme or concept that occurred in the analysis relates to the barriers to using IDDS, the benefits to the patient, and the common challenges that currently exist for patients and clinicians in the United Kingdom. One barrier was a concern from both current users of IDDS and non-users regarding the risk of infection in patients who may already be immunocompromised due to cancer treatment. Secondly, participants often believe that there is a contraindication for implantable drug delivery devices for patients with a limited life expectancy. Their minimum estimates ranged from the patient having an expected life of at least four months up to one year of life to qualify for an implantable pain device. Additionally, this approach neglects to consider the evidence that the life expectancy of patients with an implanted intrathecal device may improve due to a corresponding reduction in the requirement for systemic strong analgesic drugs. The difference in each participant's expectations is warranted as the current guidelines do not present a standard life expectancy nor do many of the randomized controlled trials assess strictly patients at the end of life. According to the NHS Clinical Commissioning Policy for intrathecal pumps, “*Research in cancer pain and ITDD is difficult as there are ethical issues around double-blind randomised trials in a group of patients that are suffering significantly with poor quality of life due to severe pain and may have limited survival. It may also be unethical to subject them to another randomized controlled trial while high-quality evidence may already be available as in case of this therapy* (2)*”*.

As hypothesized, participants who are currently treating patients with IDDS have differing perceptions of their potential benefits compared to those who do not currently treat with this technology. Clinicians who actively treat using IDDS present many clinical benefits of using infusion devices such as fewer opioids, quicker titration, less side effects. Those who are not currently using this treatment feel that infusion devices are only suited for a unique patient type, which includes those with curative cancer.

In general, one of the challenges to choosing IDDS as a treatment option continues to be the participants’ experience with various pain management techniques and the best practices associated with them. Even those who frequently choose to treat their patients using IDDS, feel there is a responsibility to weigh the benefits vs. the possible risks in terms of the patient undergoing implantation procedure.

Due to the lack of availability in some locations, clinicians were unsure of the process to refer patients. They felt that having greater access to information would improve awareness and offer intrathecal delivery more frequently as an option for refractory pain.

Another challenge is determining the specialty that is responsible for the patient pathway. There appears to be a degree of disconnect between the treating oncologists, pain clinicians, and the clinicians who would be implanting the device. The path from identifying the need for further pain management, referral to implanter, and personnel managing the patient after the implant is performed crosses multiple specialties and is not always clearly defined.

### Technical and administrative

In addition to the clinical decision-making used in assessing IDDS, there is also an indirect connection to technical and administrative perceptions. The impact of the cost of IDDS compared to other treatment options, the logistical aspects of managing devices after implant—namely dose titration, post-MRI checks and refilling, and the limitations of the current guidelines are assigned to this domain. Even though the cost of care should not be a primary influence in clinical decision-making, clinicians practicing in resource-limited health systems do need to have an awareness of cost when determining the next treatment steps for patients. From an economic perspective, participants believed that if the implant reduced the need for follow-up visits or possibly hospitalization to optimise pain management, then there would be economic value. In contrast, institutions that do not currently offer implantable pain devices may face delays in procurement, thus delaying the patient's care. The authors believe though a few studies have been conducted comparing the cost of TDD and conventional medical therapy, it shows that after 6 months of implant, it is cost-neutral, but this US-based study cannot be extrapolated to the UK and its health service (24).

More economic data needs to be produced to prove increased quality of life. “Other non-cancer patients of mine that have had this procedure whom we are waiting to get funding approval for to replace their pumps that have to go back onto oral opioids and being less mobile and not able to go to work. Then there is such a stark difference when they get back onto their intrathecal pumps. With the intrathecal pump, there are fewer side effects, and their cognitive functions improve”. —Palliative Care Consultant

After the IDDS is implanted, there is also a need to have continued care for the patient. Personnel will require training on how to manage the devices immediately after the procedure and on an ongoing basis. Those participants who have experience with these pumps stated that they are much smaller than external devices, they do not need to be refilled as often, and they foresee them getting even smaller or more streamlined in the future. According to one neurosurgeon participant: “*Overall, the intrathecal pump is a better choice over an external pump, because the external pump needs more maintenance, more refills and there are more attachments so there could be more complications”.* The participant group, however, unanimously agreed that the device vendor plays a big role in the training and maintenance of the IDDS.

### Patient-centered care

The final theme or concept identified in this research encompasses patient experience by defining the care pathway, treatment strategies, and considering the patient's physical and emotional health. Due to the involvement of multiple specialties, the pathway for refractory pain management is not as clearly defined as for other diagnoses. The initial decision to consider the implantation of a device can be made by a number of specialties including oncology, palliative care, pain management, or neurosurgery. Additionally, the ongoing maintenance of the pump may commonly be managed by a different team to that which implants it.

When determining a treatment approach, the team managing the patient's care need to first outline the strategy of controlling pain vs. controlling the cancer resulting in pain. The participants determined that the top criteria for IDDS eligibility are the location of the tumor (*n* = 10), the patient's prognosis (*n* = 8), the patient's response to other treatments (*n* = 4), and patients who are non-curative or in the end stages of cancer (*n* = 3). Pain pumps are more accepted for younger patients with a long or painful prognosis. If the cancer team are not interventionalists, they may be less likely to suggest an implant.

Although pain physicians are the most important decision maker when considered in the broader healthcare team (*n* = 13) with oncologists being the second most mentioned overall (*n* = 11), patients are the main decision maker throughout the treatment pathway (*n* = 17). It is fundamentally important that patients are involved with strategies around any treatment or pain control options, especially when implanting a device such as an IDDS. Most patients are unaware of the options for advanced pain management available to them, therefore the care team should outline these options in detail. In addition to the goal of reducing pain, some patients may be concerned about the psychological impact of having a foreign body implanted, while others may be concerned about body dysmorphia. The participants who routinely use IDDS for their patients’ pain management state that they are surprised to see how small the device is, and the benefit outweighs any perceived challenges.

### Summary of the interviews

Pain that is resistant to standard pharmacological treatment is a significant problem for cancer patients, severely affecting their quality of life. Refractory pain, which does not respond to conventional medications, is experienced by 10%–20% of cancer patients. This study focuses on intrathecal pain pumps as an intervention for managing refractory pain in cancer patients in the United Kingdom. Despite being an effective option, many UK clinicians are unaware of the benefits and the process of recommending IDDSs for their patients.

The findings of this qualitative study are categorized into four themes: Education, Barriers & Benefits, Technical & Administrative, and Patient-Centered Care. The education theme highlights the need for more knowledge and awareness of IDDS among healthcare professionals. Barriers and benefits associated with IDDS use are explored which include concerns relating to infection risk and suitability for patients with short life expectancy. The technical and administrative theme addresses cost considerations, device management, and the need for improved guidelines. Patient-centered care emphasizes the importance of involving patients in decision-making and considering their physical and emotional well-being throughout the treatment pathway.

## Discussion

The findings of this study highlight several unmet needs related to the management of refractory pain in cancer patients. These have important implications for healthcare providers, facilities, specialists, patients, and medical technology manufacturers. One significant unmet need is the development of more expansive guidelines that span multiple specialties involved in the care of cancer patients, including oncology, pain management and palliative care. The current guidelines are often focused on pain management or medication dosing rather than the specific route and device used, such as intrathecal drug delivery systems (IDDS). Comprehensive guidelines that address the use of IDDS and provide clear recommendations for its implementation would be valuable in promoting consistent and evidence-based care.

This study reveals a significant gap in the knowledge and awareness of intrathecal drug delivery systems (IDDS) among healthcare professionals. This limited understanding about the benefits and appropriate use of IDDS underscores the need for comprehensive education initiatives. Educating healthcare providers about the mechanism of IDDS, its benefits, patient selection criteria, and the implantation process is essential to ensure that patients with refractory pain receive appropriate and timely treatment. Implementing educational programs that target various specialties, such as oncology, neurosurgery, pain management, and palliative care, can improve the awareness and adoption of IDDS as a viable pain management option.

The identification and definition of refractory pain play a pivotal role in determining when to consider using IDDS and this study highlights the varying criteria among clinicians when attempting to define refractory pain. Collaborative efforts among healthcare professionals are necessary to establish standardized criteria for assessing refractory pain and identifying patients who could benefit from IDDS. The debate around the appropriate life expectancy for patients eligible for IDDS also underscores the need for clear guidelines that consider both clinical and ethical aspects.

The study emphasizes the existing gaps in the provision of guidelines that are specifically tailored to intrathecal pain management. Current guidelines primarily focus on systemic pharmacological interventions and lack comprehensive recommendations for IDDS use. Collaborative efforts involving national and international pain management organizations should be directed toward developing evidence-based guidelines that address patient selection, implantation procedures, follow-up care, and patient outcomes. Additionally, healthcare policymakers and regulatory bodies should consider commissioning strategies that facilitate access to IDDS for eligible patients.

The perceived benefits of IDDS highlighted in the study, including reduced opioid consumption, better pain control, and improved quality of life, underscore its potential to significantly impact patient outcomes. These findings support the argument for increased adoption of IDDS as part of comprehensive pain management strategies for cancer patients with refractory pain. In addition, a number of risks were discussed. Infection risk associated with IDDS remains a concern, particularly in immunocompromised cancer patients, but a prospective RCT of 1,403 patients demonstrates infection requiring surgical intervention is only 3.8% (25). While the participants of the study could not provide specific infection percentages, they were all apprehensive about it. The PACC guidelines highlight the importance of stringent infection prevention measures. Emphasizing proper device care, patient education, and adherence to established protocols can contribute to minimizing infection risks associated with IDDS.

Other unmet needs include the requirement for greater awareness among clinicians in all specialties, as well as among patients and their family members. Patients also have a crucial role in the management of refractory pain, and they need to be educated about the available options. Medical technology vendors also play a significant role in promoting effective pain management. They can support clinicians by providing comprehensive education on device implantation, management, and troubleshooting. Collaboration between vendors and medical/regulatory bodies can contribute to the dissemination of knowledge and the implementation of best practices in pain management. Finally, the impact of effective pain management using IDDS on the trajectory of oncological diseases should be recognized. Early and efficient pain control has the potential to improve patients’ overall well-being, enhance their ability to tolerate treatments, and positively influence disease outcomes. By incorporating IDDS as a part of comprehensive cancer care, healthcare providers can address not only the physical pain experienced by patients but also its impact on the disease trajectory and overall prognosis.

## Conclusion

In conclusion, this research clearly outlines the need for increased awareness and education about IDDS for managing refractory pain in cancer patients in the UK. The study reveals barriers, benefits, technical considerations, and the importance of patient-centered care in the implementation of IDDS in an oncology population. The findings provide valuable insights for healthcare professionals, aiming to improve pain management options and enhance the quality of life for cancer patients with refractory pain.

## Data Availability

The original contributions presented in the study are included in the article/Supplementary Material, further inquiries can be directed to the corresponding author.
